# Particle Size Distribution of Bimodal Silica Nanoparticles: A Comparison of Different Measurement Techniques

**DOI:** 10.3390/ma13143101

**Published:** 2020-07-11

**Authors:** Mohammed A. Al-Khafaji, Anikó Gaál, András Wacha, Attila Bóta, Zoltán Varga

**Affiliations:** Institute of Materials and Environmental Chemistry, Research Centre for Natural Sciences, H-1117 Budapest, Hungary; al-khafaji.mohammed@ttk.hu (M.A.A.-K.); gaal.aniko@ttk.hu (A.G.); wacha.andras@ttk.hu (A.W.); bota.attila@ttk.hu (A.B.)

**Keywords:** silica nanoparticle, size distribution, light scattering, small-angle X-ray scattering, microfluidic resistive pulse sensing

## Abstract

Silica nanoparticles (SNPs) belong to the most widely produced nanomaterials nowadays. Particle size distribution (PSD) is a key property of SNPs that needs to be accurately determined for a successful application. Many single particle and ensemble characterization methods are available for the determination of the PSD of SNPs, each having different advantages and limitations. Since most preparation protocols for SNPs can yield bimodal or heterogeneous PSDs, the capability of a given method to resolve bimodal PSD is of great importance. In this work, four different methods, namely transmission electron microscopy (TEM), dynamic light scattering (DLS), microfluidic resistive pulse sensing (MRPS) and small-angle X-ray scattering (SAXS) were used to characterize three different, inherently bimodal SNP samples. We found that DLS is unsuitable to resolve bimodal PSDs, while MRPS has proven to be an accurate single-particle size and concentration characterization method, although it is limited to sizes above 50 nm. SAXS was found to be the only method which provided statistically significant description of the bimodal PSDs. However, the analysis of SAXS curves becomes an ill-posed inverse mathematical problem for broad size distributions, therefore the use of orthogonal techniques is required for the reliable description of the PSD of SNPs.

## 1. Introduction

Silica nanoparticles (SNPs) represent one of the most widely utilized engineered nanomaterials. Due to the versatile properties of SNPs, their application is widespread in many fields ranging from chemical and food industry, through environmental sciences, to medical applications [1,2,3,4,5]. The most commonly used process for the preparation of SNPs is based on the controlled hydrolysis and condensation of alkoxysilanes [1]. Controlled growth of monodisperse SNPs in the 50 nm to 2000 nm size range in water-ethanol solution using ammonia as a base catalyst was first reported by Stöber et al. [6]. This method was further optimized and widely used nowadays [7,8]. In this method, the particle growth rate is limited by the hydrolysis of alkoxysilane molecules and proceeds through the surface condensation of hydrolyzed monomers or small oligomers. An analogous method was introduced by Yokoi et al. [9], which uses L-lysine instead of ammonia as the base catalyst and dosing the alkoxysilane from a top organic layer (octane) to ensure slow saturation of the aqueous phase. This procedure was further developed by Hartlen et al. [10] using L-arginine as base catalyst and cyclohexane as top organic layer. This procedure yields highly monodisperse SNPs and offers the possibility of growing the particles further step-by-step. The fast hydrolysis of alkoxysilane monomers or the presence of salts or charged byproducts, which causes the screening of electrostatic interactions, may result in the formation of bimodal dispersions when these synthesis protocols are used [11,12].

In most applications of SNPs, particle size distribution (PSD), shape, charge and stability are the most important physicochemical properties that govern the applicability of SNPs. Transmission electron microscopy (TEM) is the gold standard method to characterize the size and morphology of nanoparticles [13]. Though TEM can provide accurate characterization of monodisperse particle distributions, its accuracy is deteriorated for bimodal and heterogeneous distributions due to the fact that it is a single-particle characterization method and the number of observed particles is limited to few hundreds or thousands at most. Dynamic light scattering (DLS) is also one of the most frequently used methods to measure the PSD of nanoparticles. The low instrumental cost and fast measurement procedure of DLS makes this technique very popular. As an indirect, ensemble technique based on the light scattering of particles, DLS is biased towards larger sizes and has limited capabilities of resolving bimodal and heterogeneous PSDs [14,15,16], although monodisperse PSDs can be accurately determined by DLS [17,18,19]. Particle tracking analysis (also known as nanoparticle tracking analysis, PTA or NTA, respectively) and resistive pulse sensing (RPS) are emerging single particle detection techniques for measuring the PSD and the concentration of nanoparticles [20,21]. Microfluidic resistive pulse sensing (MRPS) is a novel realization of the Coulter-principle on a microfluidic cartridge, which enables to count a statistically significant number of particles (in the range of 10,000) and provides the number concentration of the particles as well [22].

Many comparative studies were published recently utilizing two or more techniques for the characterization of the PSD of soft and hard nanoparticles [15,20,23,24,25,26,27,28,29,30]. In summary, two main conclusions can be drawn from these comparative studies. The first is that methodological biases and the fact that different methods measure different physical quantities already result in variation in the measured diameters for monodisperse PSDs. An example for this is demonstrated by Cascio et al., who characterized silver nanoparticles in the 20 nm to 100 nm size range by using various techniques and found 41.7 nm average diameter by DLS (uncertainty not provided), while 32.3 ± 3.2 nm by TEM [23]. In our previous study, we have shown, by measuring monodisperse phospholipid vesicle systems with different particle characterization techniques, that the average hydrodynamic diameter by PTA and DLS exceeds the size obtained by non-optical methods by 30 nm to 40 nm [30]. However, the reverse trend can also be observed in some cases; e.g., Tuoriniemi et al. obtained smaller hydrodynamic diameters for SNPs (39.1 ± 2.2 nm and 41.0 ± 1.2 nm by scanning electron microscopy vs. 36.5 ± 0.3 nm and 37.3 ± 0.3 nm by DLS, respectively), and explained the observations by a permeable gel layer on the surface of the particles, which alters the particles’ diffusion constant [24]. The other common observation is that scattering methods overestimate the particle size and are unable to resolve smaller particles in case of multimodal distributions. For example, Anderson et al. studied a mixture of polystyrene particles of various sizes (220 nm, 330 nm and 410 nm diameter), and concluded that light scattering methods such as DLS and PTA cannot resolve three different populations [15]. Similarly, Mahl et al. found that only the large particle fraction can be detected by DLS and PTA in the 1:1 mixture of 70 nm silver and 15 nm gold nanoparticles [14].

Small-angle X-ray scattering (SAXS) is another nanoparticle characterization method which is capable of traceable size determination, which means that the measurement result can be related to the SI unit “meter” through an unbroken chain of comparisons with known uncertainties. Although SAXS is an ensemble method based on the elastic scattering of X-ray photons, some examples demonstrate that it is capable of resolving bimodal PSDs with the least squares fitting of the measured scattering curve by using an appropriate model function [31,32]. Gleber et al. studied the capabilities of SAXS for the characterization of bimodal poly(methyl methacrylate) particles prepared by mixing particles with nominal average diameters of 109 nm and 192 nm. SAXS was capable of resolving both size fractions with 0.7% relative uncertainty by using a model-fitting procedure assuming Gaussian distributions [31]. At the same time, if the size distribution is broad or if the nanoparticles have inner structure, the fitting of the scattering curves could become an ill-posed inverse mathematical problem, due to the large number of model parameters. This means that an unequivocal solution for calculating the size distribution from the scattering curve at sufficient significance levels does not exist. An example of the latter is the smearing of the scattering curves which can be caused by broadening of the size distribution but also by the presence of elongated ellipsoidal particles [33].

It should also be mentioned that nanoparticle systems with bimodal PSDs which were employed in studies that compare various size characterization methods were obtained by mixing two monodisperse particle samples. Intrinsically bimodal dispersions are rarely studied, despite the ability of the most common synthesis protocols of SNPs to yield bimodal PSDs. One exception of the latter is the study of Kestens et al. [34], which describes the development of the ERM-FD102 certified reference material (Institute for Reference Materials and Measurements, Joint Research Centre, European Commission) for quality control of nanoparticle size analysis methods. ERM-FD102 is a bimodal SNP material which consists of two size classes of (18.2 ± 1.6) nm and (84 ± 2.1) nm (number-weighted modal area-equivalent diameter values as determined by TEM ± expanded uncertainty). SAXS was used among other characterization methods in this study, but the uniqueness of the model fitting procedure to obtain the size distribution from the scattering curves was not investigated.

The main goal of this paper is to compare several particle size characterization methods, namely TEM, DLS, MRPS and SAXS, for the characterization of PSDs of intrinsically bimodal SNPs. For this purpose, three different intrinsically bimodal SNP samples were prepared and characterized with the above-mentioned methods. Special emphasis is given to the limitations of the compared methods, and especially to the reliability of fitting-based interpretation of the scattering curves in SAXS for SNPs with bimodal PSDs.

## 2. Materials and Methods

### 2.1. Preparation of Silica Nanoparticles

Tetraethylorthosilicate (TEOS, 78-10-4, puriss., 99% (GC), Sigma-Aldrich, Budapest, Hungary), cyclohexane (110-82-7, Guaranteed Reagent, 99.99%, Lach-Ner, Neratovice, Czech Republic), L-arginine (74-79-3, reagent grade, ≥ 98% (TLC), Sigma-Aldrich, Budapest, Hungary), ethyl alcohol (64-17-5, ≥99.8%, VWR International, Budapest, Hungary), and ammonium hydroxide solution (NH_4_OH, 1336-21-6, 25% (*v*/*v*), Reanal, Budapest, Hungary) were used as received without any further purification. Two methods were used to synthesize bimodal silica nanoparticles, which are described in detail below.

#### 2.1.1. Preparation of M015 and M0171 Bimodal Silica SNPs

In the first method, SNPs with different particle sizes were prepared by a layer-by-layer growth method using L-arginine as basic catalyst adopted from Koike et al. [35] with slight modifications. For the first bimodal SNP sample (hereinafter denoted as M015), 18.4 mg of L-arginine was transferred into a 20 mL scintillation vial (WHEATON^®^, VWR International, Budapest, Hungary) containing 16 mL Milli-Q water (18.2 MΩ cm^−1^). The solution was mixed with magnetic stirring (300 rpm) using a 1 cm Teflon-coated bar. 1.184 mL TEOS was slowly added to the upper layer. The reaction was carried out with stirring at 60 ± 0.5 °C for 24 h. Next, 1 mL from the aqueous phase was transferred into a new 10 mL vial, 4 mL Milli-Q water and 2.3 mg L-arginine were added, and 1.1 mL TEOS was layered on top of the aqueous phase. The reaction was carried out with stirring (300 rpm) for 24 h at 60 ± 0.5 °C. The aqueous phase was transferred into a clean vial at the end of the synthesis.

The same protocol with modified molar ratios was used for M0171. In the first step, 9.1 mg L-arginine in 4 mL Milli-Q water with 1.1 mL TEOS layered on top of the aqueous phase was stirred (300 rpm) for 24 h at 60 ± 0.5 °C. Then, 1 mL from the aqueous phase was transferred into a new vial, diluted with 2 mL Milli-Q water. 4.6 mg of L-arginine was added to the reaction mixture and 0.824 mL TEOS was layered on top of the aqueous phase. The second step was carried out for 24 h 60 ± 0.5 °C. Both samples synthesized by this method have been used without purification.

#### 2.1.2. Preparation of SNP022 Bimodal Silica SNPs

A bimodal SNP sample with an average particle size in the 50 nm to 100 nm range was prepared according to the Stöber method [11,36,37,38]. 10 mL ethanol with 600 µL ammonium hydroxide solution (25 *v*/*v*%) was first mixed for 10 min in a 20 mL scintillation vial (WHEATON^®^, VWR International, Budapest, Hungary) and then 300 µL TEOS solution was added quickly while stirring the solution at 500 rpm. Afterward, the solution was stirred for 15 h at ambient condition (24 ± 2 °C, 1 atm). The prepared SNPs were washed by ultrafiltration using a Millipore solvent resistant stirred cell assembled with a polyethersulfone ultrafiltration disc (PBMK07610, NMWL 300 kDa, Ø76 mm, Millipore^®^, Budapest, Hungary). A volume of 5 mL prepared silica nanoparticle solution was diluted to 100 mL with ethanol (abs.) and washed 2 times with 100 mL ethanol and 2 times with 100 mL Milli-Q water. The volume of the retentate was adjusted to 25 mL in the final step.

### 2.2. Sample Characterization

#### 2.2.1. Transmission Electron Microscopy (TEM)

Morphological investigations of SNPs were carried out on a JEM 1011 (JEOL Ltd., Tokyo, Japan) transmission electron microscope operating at 80 kV. Samples were dropped and dried on 200 mesh carbon coated copper grid and images were taken with an Olympus Morada 11-megapixel camera and using the iTEM 5.0 software (Olympus, Tokyo, Japan). The magnification calibration of the instrument was checked by using ERM-FD100 certified reference material (Institute for Reference Materials and Measurements, Joint Research Centre, European Commission, Geel, Belgium). PSDs were calculated by using the ImageJ software (Version 1.46, National Institute of Mental Health, Bethesda, MD, USA). A diameter of at least 150 particles were measured for each sample and a 5 nm bin size was used to produce PSD histograms.

#### 2.2.2. Dynamic Light Scattering (DLS)

DLS measurements were performed by using a W130i Dynamic Light Scattering System (AvidNano, High Wycombe, UK), equipped with a laser diode (λ = 660 nm) and a photodiode detector at a fixed angle of 90°. The measurements were carried out at a controlled temperature of 20 ± 0.2 °C, using low-volume disposable plastic cuvettes (UVette, Eppendorf Austria GmbH, Wien, Austria). Intensity autocorrelation functions were analyzed with the iSize 3.0 software (AvidNano, High Wycombe, UK), which utilizes a continuous I(D) distribution model. Number-weighted PSDs were calculated assuming spherical morphology with constant refractive index. In principle, DLS is an absolute method, i.e., there is no need for instrument response calibration. Nanobead NIST Traceable Particle Size Standard with 90 nm nominal diameter (Polysciences, Inc., Warrington, PA, USA) was used for quality control measurements.

#### 2.2.3. Microfluidic Resistive Pulse Sensing (MRPS)

MRPS measurements were performed with a nCS1 instrument (Spectradyne LLC, Torrance, CA, USA). SNP samples were 100-fold and 1000-fold diluted in aqueous solution of 150 mmol·L^−1^ NaCl and 1 *w*/*v*% Tween 20 (9005-64-5, Sigma-Aldrich, Budapest, Hungary) filtered through an Amicon Ultra, 100 kDa MWCO membrane filter (Sigma-Aldrich, Budapest, Hungary) for SNP022 and M0171 samples, respectively. Factory calibrated TS-300 cartridge with 50 nm to 300 nm measurement range and TS-400 cartridge with 65 nm to 400 nm measurement range were used for M0171 and SNP022 samples, respectively. Since the particle size of both fractions of the M015 was below the lower detection limit of the TS-300 cartridge, only the M0171 and SNP022 samples were characterized by this method. Nanobead NIST Traceable Particle Size Standard with 90 nm nominal diameter (Polysciences, Inc., Warrington, PA, USA) was used for size calibration.

#### 2.2.4. Small Angle X-Ray Scattering (SAXS)

SAXS measurements have been performed on CREDO, our in-house developed apparatus [39]. After a fourfold dilution with their respective solvents, the nanoparticle samples were filled into borosilicate glass capillaries (WJM Glas GmbH, Berlin, Germany) of 1.5 mm outer diameter and 0.01 mm nominal wall thickness. X-rays were generated using a GeniX^3D^ Cu ULD integrated beam delivery system using a FOX^3D^ parabolic Si/W graded multilayer mirror (Xenocs SA, Sassenage, France). The beam was collimated using a three-pinhole setup to a 1.3 mm circle at the sample position [40]. Scattering patterns were recorded using a Pilatus-300k CMOS hybrid pixel position sensitive detector [41,42,43]. Instrument-independent calibration of the angular scale was performed using an in-house calibrated silver behenate specimen [44,45]. The angular dependence of the scattering patterns and curves was expressed in terms of q, the scattering variable (being the modulus of the momentum transfer vector, related to the scattering angle as q=4πsin(θ)/λ where 2θ is the scattering angle and λ=0.154 nm is the X-ray wavelength). The intensity scale was calibrated into absolute units of differential scattering cross-section using a piece of glassy carbon obtained from Jan Ilavsky (Advanced Photon Source, Chicago, IL, USA), re-calibrated against pure water [46]. In order to assess sample stability throughout the measurement and to be able to reduce artefacts from cosmic radiation and external background, several 5 min long exposures were made on each sample, with frequent re-measuring of the calibrants and external and internal background signals. After exposure, the scattering patterns were corrected for background signals, X-ray absorption on the sample and geometrical distortions using the standard data reduction routine implemented in the instrument control software. After removing images affected by artifacts, the remaining ones were averaged for each sample. Scattering curves were produced by azimuthally averaging the final scattering patterns. Solvent background was also subtracted from each scattering curve. Quality assurance of the CREDO instrument was carried out by using ERM-FD100 certified reference material (Institute for Reference Materials and Measurements, Joint Research Centre, European Commission, Geel, Belgium).

The measured scattering curves were analyzed by an in-house developed data analysis software utilizing least-squares model fitting algorithm written in Python scripting language. The applied model functions are presented in detail below. In general, the scattered intensity of a polydisperse system of spherical, solid nanoparticles of homogeneous electron density is

(1) $$I\left(q\right)=r_{e}^{2}{\left(\Delta \rho \right)}^{2}n\int_{0}^{\infty }f\left(D\right)F^{2}\left(q,D\right)dD,$$

where $r_{e}$ is the classical radius of the electron, Δρ is the excess electron density of the nanoparticles against the solvent level, n is the number of particles in unit volume i.e., the concentration, f(D) is the probability density function of particles with diameter D, normalized to unit area under curve, and F(q,D) is the form factor amplitude of particles with diameter D. Practically, as the excess electron density is obtained separately and carries its own uncertainty, we used the relative scale parameter defined as $n^{\prime }=r_{e}^{2}{\left(\Delta \rho \right)}^{2}n$ for fitting the concentrations.

In this work we employ three different models for the analysis of SAXS curves. First, the scattered intensity assuming bimodal Gaussian distributions was used according to the following equation:

(2) $$I_{G2}\left(q\right)=n_{1}^{\prime }\frac{1}{\sqrt{2\pi \sigma_{1}^{2}}}\int_{0}^{\infty }e^{-\frac{{\left(D-D_{1}\right)}^{2}}{2\sigma_{1}^{2}}}F_{\mathrm{sphere}}^{2}\left(q,D\right)dD+n_{2}^{\prime }\frac{1}{\sqrt{2\pi \sigma_{2}^{2}}}\int_{0}^{\infty }e^{-\frac{{\left(D-D_{2}\right)}^{2}}{2\sigma_{2}^{2}}}F_{\mathrm{sphere}}^{2}\left(q,D\right)dD,$$

where $D_{1}$, $D_{2}$, $\sigma_{1}$, $n_{1}^{\prime }$ and $n_{2}^{\prime }$ are the mean, standard deviation and relative concentration parameters, respectively, of the two fractions and the scattering form factor amplitude of a homogeneous sphere of diameter D is

(3) $$F_{sphere}\left(q,D\right)=\frac{4\pi }{q^{3}}\left(\sin\frac{qD}{2}-\frac{qD}{2}\cos\frac{qD}{2}\right).$$

In order to assess the need of the second size fraction, the unimodal variant of the previous model was also tested:

(4) $$I_{G1}\left(q\right)=n^{\prime }\frac{1}{\sqrt{2\pi \sigma^{2}}}\int_{0}^{\infty }e^{-\frac{{\left(D-D_{0}\right)}^{2}}{2\sigma^{2}}}F_{\mathrm{sphere}}^{2}\left(q,D\right)dD,$$

where the normal distribution is located at $D_{0}$ and its standard deviation is σ.

As nucleation and growth processes typically result in log-normal size distributions [47], the log-normal variants of the above models:

(5) $$I_{L-N1}\left(q\right)=n^{\prime }\frac{1}{\sqrt{2\pi \sigma^{2}}}\overset{\infty }{\underset{0}{\int }}\frac{1}{D}e^{-\frac{{\left(\ln D-\mu_{0}\right)}^{2}}{2\sigma^{2}}}F_{\mathrm{sphere}}^{2}\left(q,D\right)dD$$

and

(6) $$I_{L-N2}\left(q\right)={n_{1}}^{\prime }\frac{1}{\sqrt{2\pi \sigma_{1}^{2}}}\overset{\infty }{\underset{0}{\int }}\frac{1}{D}e^{-\frac{{\left(\ln D-\mu_{1}\right)}^{2}}{2\sigma_{1}^{2}}}F_{\mathrm{sphere}}^{2}\left(q,D\right)dD+{n_{2}}^{\prime }\frac{1}{\sqrt{2\pi \sigma_{2}^{2}}}\overset{\infty }{\underset{0}{\int }}\frac{1}{D}e^{-\frac{{\left(\ln D-\mu_{2}\right)}^{2}}{2\sigma_{2}^{2}}}F_{\mathrm{sphere}}^{2}\left(q,D\right)dD$$

were also tried. Here $\mu_{0}$, $\mu_{1}$, $\mu_{2}$, σ, $\sigma_{1}$ and $\sigma_{2}$ correspond to the means and standard deviations of the underlying normal distributions.

Finally, a core-shell model was also tested:

(7) $$I_{\mathrm{CS}}\left(q\right)=n^{\prime }\frac{1}{\sqrt{2\pi \sigma^{2}}}\overset{\infty }{\underset{0}{\int }}e^{-\frac{{\left(D-D_{0}\right)}^{2}}{2\sigma^{2}}}F_{\mathrm{CS}}^{2}\left(q,D,\tau ,\delta \rho \right)dD,$$

where the scattering form factor amplitude of a core-shell particle is

(8) $$F_{\mathrm{CS}}\left(q,D,\tau ,\delta \rho \right)=\delta \rho F_{\mathrm{sphere}}\left(q,D\cdot \tau \right)+\left(1-\delta \rho \right)F_{\mathrm{sphere}}\left(q,D\right).$$

Here, τ is the ratio of the shell thickness and the diameter of the particle and δρ is the scaled electron density of the shell if the core is unity and the solvent level is zero.

To compare the goodness of fits of the models, two commonly used quantities have been calculated. The first one is the reduced *χ*^2^ statistics:

(9) $$\chi_{\mathrm{red}.}^{2}=\frac{1}{DoF}\underset{k}{\sum }\frac{{\left(I_{\mathrm{fitted}}\left(q_{k}\right)-I_{\mathrm{measured}}\left(q_{k}\right)\right)}^{2}}{\Delta^{2}I\left(q_{k}\right)},$$

where *DoF* is the number of degrees of freedom and $\Delta I\left(q_{k}\right)$ is the propagated error of the measured scattering curve at $q_{k}$, as obtained by the data reduction software.

The other one is the adjusted coefficient of determination by Theil ($R_{\mathrm{adj}}^{2}$):

(10) $$R_{\mathrm{adj}.}^{2}=1-\frac{\sum_{k}\frac{{\left(I_{\mathrm{fitted}}\left(q_{k}\right)-I_{\mathrm{measured}}\left(q_{k}\right)\right)}^{2}}{\Delta^{2}I\left(q_{k}\right)}/\left(DoF-1\right)}{\sum_{k}\frac{{\left(I_{\mathrm{measured}}\left(q_{k}\right)-\langle I_{measured}\left(q_{j}\right)\rangle_{j}\right)}^{2}}{\Delta^{2}I\left(q_{k}\right)}/\left(N-1\right)}$$

## 3. Results and Discussion

**Figure 1:**
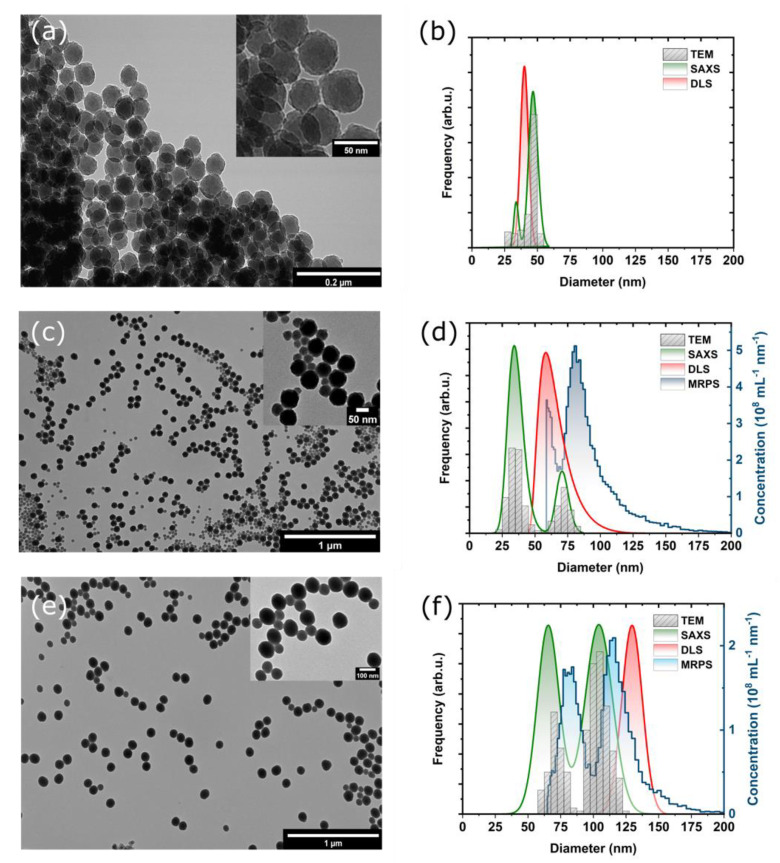
Morphological characterization and particle size distributions (PSDs) of bimodal silica nanoparticles. (**a**,**c**,**e**) Transmission electron microscopic (TEM) images of M015, M0171 and SNP022 samples, respectively. (**b**,**d**,**f**) PSDs as determined by TEM, small-angle X-ray scattering (SAXS), dynamic light scattering (DLS), and microfluidic resistive pulse sensing (MRPS) for M015, M0171 and SNP022 samples, respectively.

In this work, we investigated the capabilities of various methods for the determination of the PSD of inherently bimodal SNPs. Figure 1 shows the TEM images as well as the PSDs of the three bimodal SNP samples as determined by TEM, DLS, MRPS and SAXS.

TEM images of the prepared SNPs shown in Figure 1a,c,e reveal that all samples are bimodal. Fitting Gaussian functions to the histograms (Figure S1) enabled to calculate of the mean diameter and standard deviation (SD) values for all samples, which is summarized in Table 1. In the case of the M015 sample, the first fraction appears as a shoulder to the main fraction centered at ca. 47 nm, but two well-separated, discrete size fractions can be identified in the case of the other two samples. It should be noted that the apparent aggregation of SNPs observable on the TEM images can be attributed to the drying process during the deposition of the samples onto the TEM grids. If such aggregation would occur in solution, it would be clearly noticeable from DLS and SAXS measurements.

In contrast, DLS measurements yielded unimodal PSDs for all investigated samples, despite that the employed method for the evaluation of the intensity autocorrelation function is capable of describing multimodal distributions. Table S1 summarizes the intensity-, volume- and number-weighted PSDs determined by DLS, and the number-weighted PSDs are shown in Figure 1b,d,f for the M015, M0171 and SNP022 samples, respectively. The mean diameter determined by DLS falls between the sizes of the two fractions in the case of the M015 and M0171 samples, while it gives a mean diameter value approximately 30 nm larger than that of the second size fraction (obtained by TEM) in case of the SNP022 sample.

The observation that DLS cannot resolve two particle populations is not new and can be attributed to the fact that the intensity of the scattered light depends on the particle size according to the Mie-theory. This dependence can be approximated as $I\propto D^{6}$, which implies that larger particles scatter light orders of magnitudes more intensively than smaller ones. The intensity autocorrelation function measured by DLS is therefore biased towards larger particles. These results demonstrate that DLS alone is not reliable for the quality control of the PSD of SNPs during synthesis.

PSDs of the M0171 and SNP022 samples measured by MRPS are also shown in Figure 1d,f, respectively. The lower detection limit of 50 nm of MRPS did not allow the characterization of the M015 sample with this method. The detection limit also implies that only the larger fraction can be characterized in the case of the M0171 sample. MRPS is based on the Coulter-principle, hence it is a single particle detection method. During the measurement, the particles dispersed in a conductive medium are passed through a single nanopore. Due to the displacement of electrolyte caused by the particle, an impedance pulse can be measured, the height of which is proportional to the size of the particle. Counting the pulses within a certain measurement time and knowing the flow rate enables the determination of the concentration of the particles. The mean diameter and standard deviation (SD) of fraction #2 of M0171 using a Gaussian fit were found to be 81.6 nm and 11.3 nm, respectively. For SNP022, both fractions were measurable by MRPS. 76.9 nm was obtained for the mean diameter and 10.0 nm for the SD of fraction #1, and 108.5 nm for the mean diameter and 12.8 nm for the SD were obtained for fraction #2. Mean diameter values measured by MRPS are consistently larger than those determined by TEM. The difference is 9.7 nm for fraction #2 of M0171, and 8.3 nm and 7 nm for fractions #1 and #2 of SNP022, respectively. This observation might be attributed to the adsorption of Tween 20 on the surface of the particles, which is used to reduce the surface tension of the sample in order to enable the proper wetting of the microfluidic channels. This hypothesis needs further validation, which is out of the scope of the current work. Since MRPS measures the PSD on an absolute scale, particle concentrations were also determined: $1.37\times 10^{13}$ mL^−1^ was obtained for fraction #2 of M0171, and $4.10\times 10^{11}$ mL^−1^ and $6.16\times 10^{11}$ mL^−1^ were measured for fractions #1 and #2 of SNP022, respectively.

**Figure 2:**
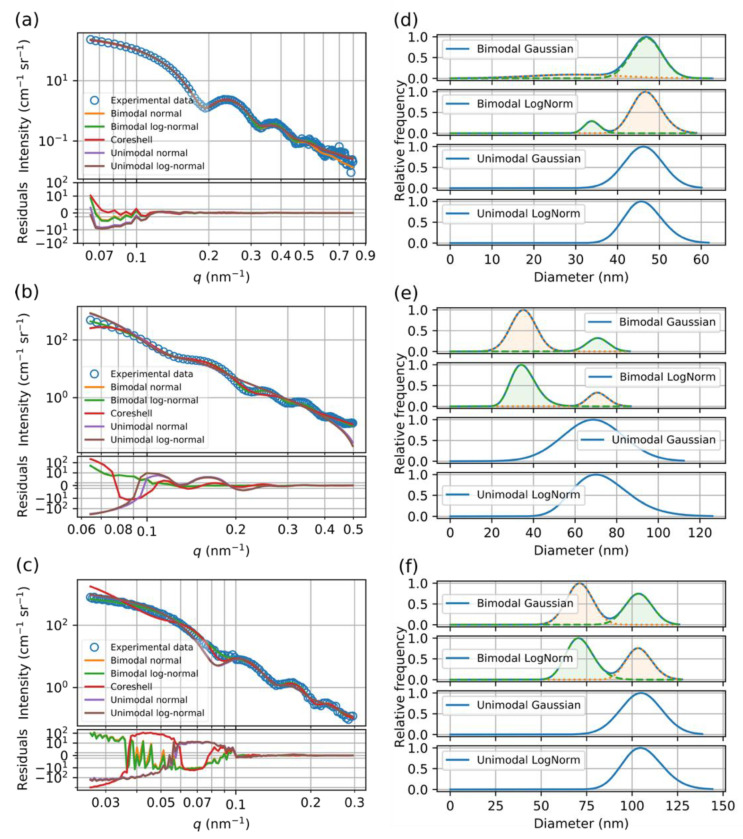
SAXS curves of (**a**) M015, (**b**) M0171 and (**c**) SNP022 samples. The experimental data (open circles) are plotted together with the fitting curves (lines) of various models. The graphs on the right show the fitted size distribution of various models for samples (**d**) M015, (**e**) M0171 and (**f**) SNPs22.

The fourth method for the characterization of the PSDs of the bimodal SNPs was SAXS, which is an ensemble method, but its applicability to determine the PSD of the mixture of two sufficiently monodisperse particles was already demonstrated [31]. In SAXS, the scattered intensity is measured as a function of the momentum transfer, *q*, which is directly related to the scattering angle. Structural parameters of the scattering objects, e.g., the PSD of the particles in our case, can be obtained by model fitting procedures as described in Section 2.2.4. The measured scattering curves of the investigated samples together with the best fitting curves of various models are shown in Figure 2a–c. The used model functions include unimodal and bimodal distributions using normal and log-normal functions for the different size fractions. An additional core-shell model was also tested, as it can account for the possible structural differences within the particles created by the two-step growth process used for the preparation of M015 and M0171 samples. All parameters corresponding to the best fitting model functions are summarized in Table S2.

Even just looking at the SAXS curves in Figure 2 reveals that the decaying, oscillatory part of the scattering curve is governed by at least two modulations, as the first minimum is broader and shallower than the subsequent ones, indicating that more than one size fractions are required to appropriately describe the sample. The numerical results in Table 2 corroborate that bimodal models fit the measured curves much better than unimodal ones in terms of the reduced *χ*^2^ goodness of fit parameter. Additionally, log-normal size distributions fit better than normal distributions, probably because they better describe the underlying growth process. Another, general feature observed in Table 2 is that, because the scattering power of an object is proportional to the 6th power of its linear size, the larger size fraction dominates the scattering curve, making the determination of the smaller size fraction more uncertain. Finally, as the core-shell model was not able to provide satisfactory fit to the scattering curves, this alternative model can be ruled out.

The best fitting bimodal PSDs with log-normal size distributions obtained by SAXS are also shown in Figure 1b,d,f. It can be concluded from the comparison to the PSDs determined by other methods that SAXS is the only method which provides statistically significant description of the bimodal PSDs.

**Figure 3:**
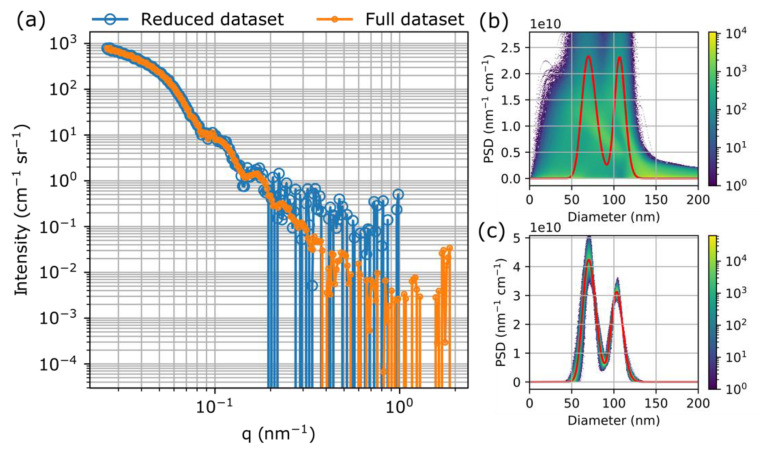
(**a**) Scattering curves of the SNP022 sample measured at a reduced and an extended (full) q-range and (**b**,**c**) the corresponding particle size distributions (PSDs) together with the heat map of possible PSDs calculated by taking the uncertainty and covariance of fitted parameters into account.

As it can be observed on the SAXS curves shown in Figure 2, the *q*-range was adjusted to the size of the particles, i.e., the first, so-called Guinier-region as well as the oscillatory part of the scattering curves are covered for all the three samples. To demonstrate the importance of the proper selection of the *q*-range, Figure 3a shows the scattering curves of the SNP022 sample measured at a reduced and an extended *q*-range. The reduced *q*-range was obtained by using only one sample-to-detector distance, while the extended *q*-range was obtained by combining the scattering curves measured at a shorter and a longer sample-to-detector distance. Figure 3b,c show bimodal log-normal PSDs corresponding to the best fitting model functions using these *q*-ranges together with a heat map of possible PSDs calculated by taking the uncertainty and covariance of fitted parameters into account. To calculate these maps, first 10,000 samples were taken from a correlated, multivariate normal distribution where the vector of mean values contained the fitted values of the parameters and the covariance matrix was that of the fit parameters. For each random sample, the corresponding size distribution was calculated on a predefined range of particle diameters. At each diameter value, histograms were made of the 10,000 different distribution function values, which were subsequently used as columns for the heat map. The PSD corresponding to the best fitting parameter values (Table S3) is also plotted over the map. The same representation of possible PSDs for M015 and M017 samples are shown in Figure S2.

Comparison of the heat maps of the PSDs from the scattering curves measured at the reduced and the extended *q*-ranges clearly indicate that the uncertainty of the resulting PSDs due to the model fitting procedure is significantly higher for the scattering curve measured at the reduced *q*-range. In both cases, the bimodal function gives a better fit than the unimodal function, but the parameters of the PSD with acceptable uncertainty can only be obtained from the curve measured at the optimal, extended *q*-range.

The concentration of the particles can also be obtained from the model fitting to the SAXS data if the measured scattered intensity is expressed in absolute units of differential scattering cross-section. The theoretical intensity formula contains a multiplication factor in the form of ${\left(\Delta \rho \right)}^{2}n$ where Δρ is the electron density contrast between the particles and the solvent and *n* is the number concentration of the particles. This implies that these quantities cannot be determined simultaneously from a single SAXS measurement. In our evaluation procedure we used an approximate value of 2 g/cm^3^ for the density Δρ. The obtained concentration values are also indicated in Table 2. There is a remarkable agreement between SAXS and MRPS for the concentration of both size fractions of SNP022, while the concentration of fraction #2 of M0171 is found significantly smaller by MRPS than by SAXS. These differences might be attributed to the different preparation procedures of the M0171 and SNP022 particles, but due to the above-mentioned assumptions, concentration values obtained by SAXS serve only as estimates. For a more reliable evaluation an independent determination of the density of the particles might be carried out, e.g., by differential centrifugal sedimentation or by applying continuous solvent contrast variation in SAXS.

## 4. Conclusions

Well-known (TEM, DLS) and less widespread methods (SAXS, MRPS) were used to characterize the PSD of three, intrinsically bimodal SNP samples, all with expected sizes below 100 nm. The analysis of the PSDs by TEM clearly revealed that all samples have bimodal PSDs, even though the used procedures are reported to result in monodisperse particles. DLS on the other hand failed to resolve the different size fractions in all three cases. MRPS could only detect size fractions larger than 50 nm, but above this threshold it could resolve bimodal PSDs and provided statistically relevant distributions with absolute concentration values based on counting > 10,000 particles. SAXS was found to be the only method which provided statistically significant description of the bimodal PSDs for all three investigated samples. The comparison of different models for the interpretation of the SAXS curves demonstrated that special attention should be paid to the uniqueness of the fitting result, and instrumental parameters such as the covered *q*-range should be carefully chosen. In conclusion, (light) scattering methods can only be used with care for the routine characterization of the PSD of SNPs, and the use of the combination of orthogonal techniques, such as TEM or MRPS to confirm the validity of the used model in the evaluation of the SAXS curves is necessary for an accurate size determination.

## Figures and Tables

**Table 1:** Mean diameter and standard deviation (SD) values of the PSDs of each size fractions of the investigated bimodal SNPs obtained by TEM. Indicated standard error values were obtained from least-squares fitting of Gaussian functions.

Sample	Fraction	TEM	
		Mean Diameter (nm)	SD (nm)
**M 015**	#1	34.5 ± 2.0	14.1 ± 4.3
	#2	47.1 ± 0.2	5.0 ± 0.2
**M 0171**	#1	34.7 ± 0.1	10.1 ± 0.3
	#2	72.0 ± 0.3	10.3 ± 0.6
**SNP022**	#1	68.8 ± 0.8	11.4 ± 2.0
	#2	101.6 ± 0.5	14.7 ± 1.3

**Table 2:** Mode and scatter parameters as well as concentrations of the size fractions of the nanoparticle systems determined by SAXS assuming uni- and bimodal normal and log-normal size distributions. Values corresponding to the best fitting bimodal PSDs with log-normal size distributions are highlighted in bold typeface. Indicated standard error values were obtained from least-squares fitting procedures.

Model	Fraction	Parameter	M015	M0171	SNP022
bimodal normal	#1	mode (nm)	46.9 ± 0.1	70.84 ± 0.03	103.5 ± 0.3
		SD (nm)	3.7 ± 0.2	5.15 ± 0.05	7.4 ± 0.5
		conc. (10^12^ mL^−1^)	80 ± 20	23 ± 5	0.42 ± 0.10
	#2	mode (nm)	30 ± 3	35.14 ± 0.06	71.1 ± 0.5
		SD (nm)	11 ± 2	6.10 ± 0.05	7.2 ± 0.8
		conc. (10^12^ mL^−1^)	22 ± 6	90 ± 20	0.5 ± 0.1
	Reduced *χ*^2^		1.49	16.61	0.37
bimodal log-normal	#1	mode (nm)	**46.7 ± 0.1**	**70.69 ± 0.03**	**103.3 ± 0.4**
		SD (nm)	**3.55 ± 0.10**	**4.76 ± 0.05**	**7.1 ± 0.5**
		conc. (10^12^ mL^−1^)	**80 ± 20**	**23 ± 5**	**0.41 ± 0.09**
	#2	mode (nm)	**33.8 ± 0.5**	**34.15 ± 0.06**	**70.5 ± 0.6**
		SD (nm)	**2 ± 1**	**6.05 ± 0.04**	**7.6 ± 0.8**
		conc. (10^12^ mL^−1^)	**12 ± 3**	**80 ± 20**	**0.6 ± 0.1**
	Reduced *χ*^2^		**0.88**	**13.37**	**0.33**
unimodal normal	#1	mode (nm)	46.17 ± 0.03	68.67 ± 0.06	104.9 ± 0.3
		SD (nm)	4.64 ± 0.03	14.53 ± 0.03	11.2 ± 0.2
		conc. (10^12^ mL^−1^)	90 ± 20	50 ± 10	0.6 ± 0.1
	Reduced *χ*^2^		2.42	697.53	3.14
unimodal log-normal	#1	mode (nm)	45.70 ± 0.03	70.05 ± 0.05	104.6 ± 0.3
		SD (nm)	4.52 ± 0.03	13.71 ± 0.03	11.0 ± 0.2
		conc. (10^12^ mL^−1^)	90 ± 20	50 ± 10	0.6 ± 0.1
	Reduced *χ*^2^		2.98	672.01	3.04

## Supplementary Materials

The following are available online at https://www.mdpi.com/1996-1944/13/14/3101/s1, Figure S1: PSD as determined by TEM for the (a) M015, (b) M0171 and (c) SNP022 samples together with fitted Gaussian functions, Table S1: Mean diameters and full width at half maximum (FWHM) values of the number-weighted, volume-weighted and intensity-weighted particle PSDs obtained by DLS, Table S2: Results of fitting the SAXS curves with different model functions, Table S3: Results of fitting the SAXS curves with the bimodal log-normal distribution model for the SNP022 sample measured at a reduced and an extended *q*-range.
