# Supplementary material for: Particle Size Distribution of Bimodal Silica Nanoparticles: A Comparison of Different Measurement Techniques
Source: Materials (Basel). 2020 Jul 11;13(14):3101. doi: 10.3390/ma13143101 (PMC7412153; doi:10.3390/ma13143101)
Supplement: Supplementary file 1 [file materials-13-03101-s001.pdf]

materials

Supplementary Materials: Particle Size Distribution of Bimodal Silica Nanoparticles: A Comparison of Different Measurement Techniques

Mohammed A. Al-Khafaji, Anikó Gaál, András Wacha, Attila Bóta and Zoltán Varga *

Institute of Materials and Environmental Chemistry, Research Centre for Natural Sciences, H-1117 Budapest, Hungary; al-khafaji.mohammed@ttk.hu (M.A.A.-K.); gaal.aniko@ttk.hu (A.G.); wacha.andras@ttk.hu (A.W.); bota.attila@ttk.hu (A.B.)

* Correspondence: varga.zoltan@ttk.hu; Tel.: +36-1-382-6568

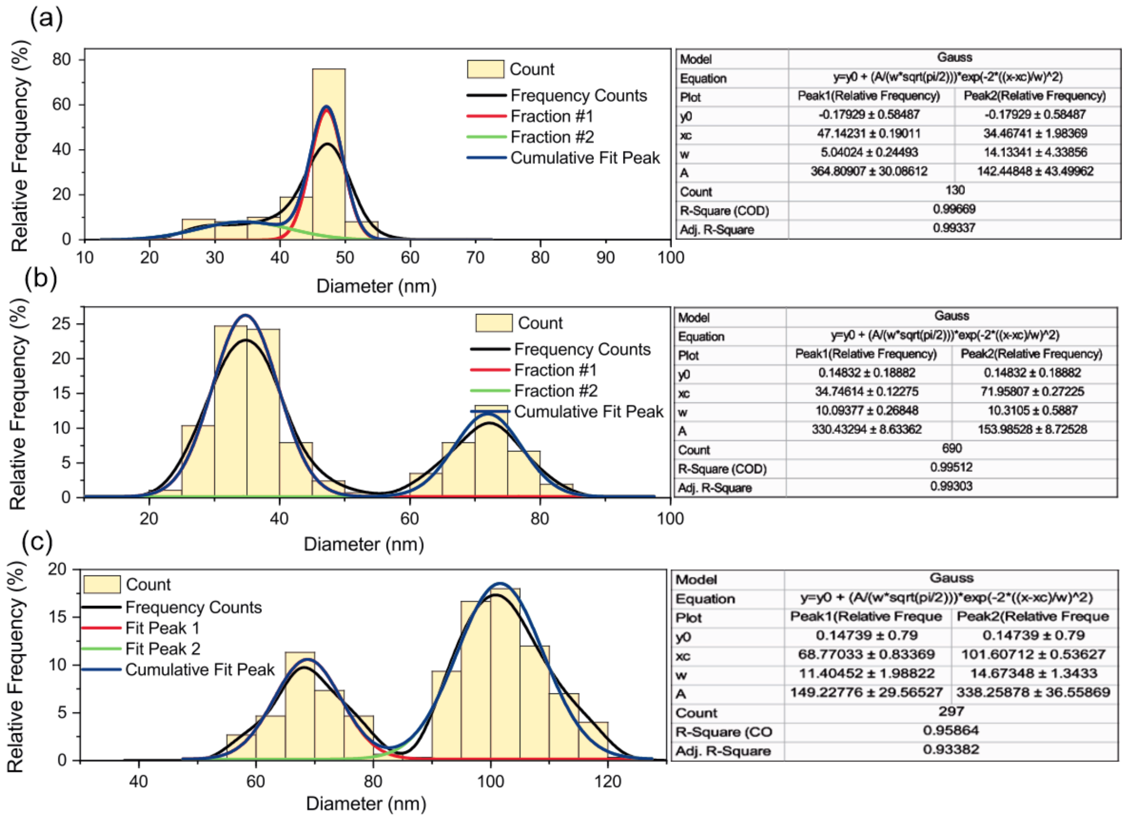

Model	Gauss	
Equation	y=y0 + (A/(w*sqrt(pi/2)))*exp(-2*((x-xc)/w)^2)	
Plot	Peak1(Relative Frequency)	Peak2(Relative Frequency)
y0	-0.17929 ± 0.58487	-0.17929 ± 0.58487
xc	47.14231 ± 0.19011	34.46741 ± 1.98369
w	5.04024 ± 0.24493	14.13341 ± 4.33856
A	364.80907 ± 30.08612	142.44848 ± 43.49962
Count	130	
R-Square (COD)	0.99669	
Adj. R-Square	0.99337	

Model	Gauss	
Equation	y=y0 + (A/(w*sqrt(pi/2)))*exp(-2*((x-xc)/w)^2)	
Plot	Peak1(Relative Frequency)	Peak2(Relative Frequency)
y0	0.14832 ± 0.18882	0.14832 ± 0.18882
xc	34.74614 ± 0.12275	71.95807 ± 0.27225
w	10.09377 ± 0.26848	10.3105 ± 0.5887
A	330.43294 ± 8.63362	153.98528 ± 8.72528
Count	690	
R-Square (COD)	0.99512	
Adj. R-Square	0.99303	

Model	Gauss	
Equation	y=y0 + (A/(w*sqrt(pi/2)))*exp(-2*((x-xc)/w)^2)	
Plot	Peak1(Relative Freque	Peak2(Relative Freque
y0	0.14739 ± 0.79	0.14739 ± 0.79
xc	68.77033 ± 0.83369	101.60712 ± 0.53627
w	11.40452 ± 1.98822	14.67348 ± 1.3433
A	149.22776 ± 29.56527	338.25878 ± 36.55869
Count	297	
R-Square (CO	0.95864	
Adj. R-Square	0.93382	

Figure S1. PSD as determined by TEM for the (**a**) M015, (**b**) M0171 and (**c**) SNP022 samples together with fitted Gaussian functions. The parameters of the best fitting model functions are summarized next to the PSDs.

(a) (b)

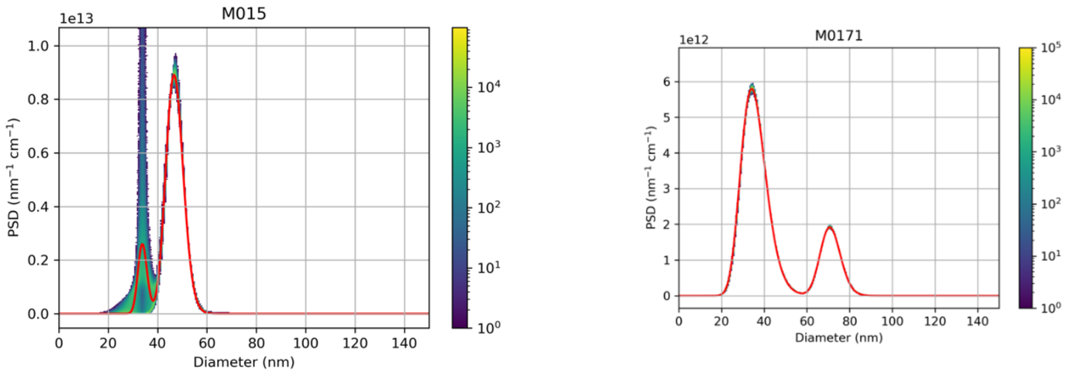

Figure S2. Best fitting model function obtained from SAXS analysis (red lines) with the heat map of possible PSDs calculated by taking the uncertainty and covariance of fitted parameters into account for the (**a**) M015 and (**b**) M0171 samples.

Table S1. Mean diameters and full width at half maximum (FWHM) values of the number-weighted, volume-weighted and intensity-weighted particle PSDs obtained by DLS. Indicated standard error values were obtained from least-squares fitting of Gaussian functions.

Sample	Diameter (number-weighted)	FWHM (number-weighted)	Diameter (volume-weighted)	FWHM (volume-weighted)	Diameter (intensity-weighted)	FWHM (intensity-weighted)
M015	40.1 ± 0.1	7.1 ± 0.2	40.9 ± 0.1	7.6 ± 0.2	41.7 ± 0.1	8.0 ± 0.1
M0171	61.2 ± 0.4	19.2 ± 1	67.8 ± 0.5	28.1 ±1.3	79.2 ± 0.5	40.2 ±1.3
SNP022	129.6 ± 0.1	18.6 ± 0.1	131.1 ± 0.1	18.5 ± 0.1	132.5 ± 0.1	18.2 ± 0.2

Table S2. (**a**) Results of fitting the SAXS curves with the unimodal normal distribution model.[1.]

Parameter	M015	M0171	SNP022
D_0 (nm)	46.17 ± 0.03	68.67 ± 0.06	104.9 ± 0.3
σ (nm)	4.64 ± 0.03	14.53 ± 0.03	11.2 ± 0.2
n (part./mL)	$2.2 \times 10^{13} \pm 4.9 \times 10^{12}$	$1.4 \times 10^{13} \pm 3.1 \times 10^{12}$	$5.9 \times 10^{11} \pm 1.3 \times 10^{11}$
C (cm^{-1} sr^{-1})	0.014 ± 0.001	−0.096 ± 0.002	0.021 ± 0.005
Red. X^2	2.42	697.53	3.14
Adj. R^2	0.999996	0.999835	1.000000
DoF	154	90	124

(**b**) Results of fitting the SAXS curves with the unimodal log-normal distribution model.[1]

Parameter	M015	M0171	SNP022
μ (ln(nm))	3.8316 ± 0.0006	4.2833 ± 0.0007	4.66 ± 0.002
σ (ln(nm))	0.0973 ± 0.0006	0.1844 ± 0.0004	0.103 ± 0.002
n (part./mL)	$2.2 \times 10^{13} \pm 4.9 \times 10^{12}$	$1.2 \times 10^{13} \pm 2.7 \times 10^{12}$	$5.7 \times 10^{11} \pm 1.3 \times 10^{11}$
C (cm^{-1} sr^{-1})	0.014 ± 0.001	−0.105 ± 0.002	0.022 ± 0.005
Red. X^2	2.98	672.01	3.04
Adj. R^2	0.999995	0.999841	1.000000
DoF	154	90	124
Mode (nm)	45.71 ± 0.03	70.05 ± 0.05	104.5 ± 0.3
SD (nm)	4.52 ± 0.03	13.71 ± 0.03	11.0 ± 0.2

(**c**) Results of fitting the SAXS curves with the bimodal normal distribution model.[1]

Parameter	SNP022	M015	M0171
D_1 (nm)	71.1 ± 0.5	30.0 ± 3.0	35.14 ± 0.06
σ_1 (nm)	7.2 ± 0.8	11.0 ± 2.0	6.1 ± 0.05
n_1 (part./mL)	$5.4 \times 10^{11} \pm 1.3 \times 10^{11}$	$5.4 \times 10^{12} \pm 1.5 \times 10^{12}$	$2.1 \times 10^{13} \pm 4.8 \times 10^{12}$
D_2 (nm)	103.5 ± 0.3	46.9 ± 0.09	70.84 ± 0.03
σ_2 (nm)	7.4 ± 0.5	3.7 ± 0.2	5.15 ± 0.05
n_2 (part./mL)	$4.2 \times 10^{11} \pm 9.7 \times 10^{10}$	$1.9 \times 10^{13} \pm 4.4 \times 10^{12}$	$5.8 \times 10^{12} \pm 1.3 \times 10^{12}$
C (cm^{-1} sr^{-1})	−0.0 ± 0.006	0.0 ± 0.001	−0.0 ± 0.002
Red. X^2	0.37	1.49	16.61
Adj. R^2	1.000000	0.999997	0.999996
DoF	121	151	87

(**d**) Results of fitting the SAXS curves with the bimodal log-normal distribution model.[1]

Parameter	M015	M0171	SNP022
μ_1 (ln(nm))	3.849 ± 0.002	4.2628 ± 0.0004	4.642 ± 0.003
σ_1 (ln(nm))	0.075 ± 0.002	0.0668 ± 0.0007	0.068 ± 0.005
n_1 (part./mL)	$2 \times 10^{13} \pm 4.4 \times 10^{12}$	$5.7 \times 10^{12} \pm 1.3 \times 10^{12}$	$4.1 \times 10^{11} \pm 9.4 \times 10^{10}$
μ_2 (ln(nm))	3.52 ± 0.02	3.559 ± 0.001	4.266 ± 0.006
σ_2 (ln(nm))	0.05 ± 0.05	0.169 ± 0.001	0.11 ± 0.01
n_2 (part./mL)	$3 \times 10^{12} \pm 7.8 \times 10^{11}$	$2.1 \times 10^{13} \pm 4.8 \times 10^{12}$	$5.7 \times 10^{11} \pm 1.4 \times 10^{11}$
C (cm^{-1} sr^{-1})	0.014 ± 0.001	0.002 ± 0.002	−0.01 ± 0.006
Red. X^2	0.88	13.37	0.33
Adj. R^2	0.999998	0.999997	1.000000
DoF	151	87	121
Mode #1 (nm)	46.7 ± 0.1	70.69 ± 0.03	103.3 ± 0.4
SD #1 (nm)	3.55 ± 0.1	4.76 ± 0.05	7.1 ± 0.5
Mode #2 (nm)	33.8 ± 0.5	34.15 ± 0.06	70.5 ± 0.6
SD #2 (nm)	2.0 ± 1.0	6.05 ± 0.04	7.6 ± 0.8

(**e**) Results of fitting the SAXS curves with the core-shell particle model.[1]

Parameter	M015	M0171	SNP022
D_0 (nm)	46.43 ± 0.04	35.91 ± 0.04	101.0 ± 0.4
σ (nm)	4.57 ± 0.03	6.66 ± 0.01	9.5 ± 0.2
n (part./mL)	$2.1 \times 10^{13} \pm 4.8 \times 10^{12}$	$2 \times 10^{13} \pm 4.6 \times 10^{12}$	$7.9 \times 10^{11} \pm 1.8 \times 10^{11}$
τ	0.25 ± 0.01	1.402 ± 0.001	0.74 ± 0.003
$\delta\rho$	−0.013 ± 0.001	−0.197 ± 0.001	0.097 ± 0.005
C (cm^{-1} sr^{-1})	0.012 ± 0.001	0.017 ± 0.002	0.001 ± 0.005
Red. X^2	1.50	102.48	0.68
Adj. R^2	0.999997	0.999976	1.000000
DoF	152	88	122

1 Indicated standard error values were obtained from least-squares fitting.

Table S3. Results of fitting the SAXS curves with the bimodal log-normal distribution model for the SNP022 sample measured at a reduced (reduced dataset) and an extended q-range (full dataset). Indicated standard error values were obtained from least-squares fitting.

Fraction	Parameter	Full Dataset	Reduced Dataset
	mode (nm)	103.3 ± 0.4	106.0 ± 9.0
**1**	scatter (nm)	7.1 ± 0.5	9.0 ± 6.0
	concentration (10^{12} mL^{-1})	0.41 ± 0.09	0.4 ± 0.3
	mode (nm)	70.5 ± 0.6	70.0 ± 10.0
**2**	scatter (nm)	7.6 ± 0.8	20.0 ± 20.0
	concentration (10^{12} mL^{-1})	0.6 ± 0.1	0.5 ± 0.4
**Goodness of fit**	Reduced χ^2	0.33	0.06

© 2020 by the authors. Submitted for possible open access publication under the terms and conditions of the Creative Commons Attribution (CC BY) license (http://creativecommons.org/licenses/by/4.0/).
